# Trends in Incidence of Cancers Associated With Obesity and Other Modifiable Risk Factors Among Women, 2001–2018

**DOI:** 10.5888/pcd20.220211

**Published:** 2023-03-30

**Authors:** Katherine R. Cotangco, Cheng-I Liao, Cortney M. Eakin, Ava Chan, Joshua Cohen, Daniel S. Kapp, John K. Chan

**Affiliations:** 1 UCLA School of Medicine, Los Angeles, California; 2 Kaohsiung Veterans General Hospital, Kaohsiung, Taiwan; 3 California Pacific Medical Center Research/Sutter Health, San Francisco, California; 4 Division of Gynecologic Oncology, Department of Surgery, City of Hope, Irvine, California; 5 Stanford School of Medicine, Stanford, California

## Abstract

We used data from the US Cancer Statistics database to determine trends in cancer incidence, stratified by age, race, and ethnicity, among women aged 20 years or older during an 18-year study period (2001–2018). We limited analysis to cancers associated with 5 modifiable risk factors: tobacco use, excess body fat, alcohol consumption, insufficient physical activity, and human papillomavirus infection. The incidence of cancers associated with obesity have risen, particularly among women aged 20 to 49 years (vs ≥50 y) and among Hispanic women. Strategies that address obesity rates in these populations may help decrease cancer risk.

**Summary**

**What is already known on this topic?**

The incidence of cancer is rising among women; 42% of cancers among men and women are associated with modifiable risk factors.

**What is added by this report?**

We used 18 years of population-based data to determine trends in the incidence of cancers that are associated with 5 modifiable risk factors. The incidence of obesity-associated cancer is rising, particularly among women aged 20 to 49 years (vs ≥50 y) and Hispanic women.

**What are the implications for public health practice?**

Our findings help advance the understanding of cancer prevention. Studies that examine the effect of obesity on cancers diagnosed among young and Hispanic women are warranted.

## Objective

Cancer incidence rates are rising and are projected to increase by 30% among US women by 2030 (1). An estimated 934,870 new cases of cancer among women are expected in 2022, with breast, lung, and colorectal cancer accounting for 51% of new cases (2).

Approximately 42% of cancers among men and women are associated with modifiable risk factors, including tobacco use, excess body fat, alcohol consumption, insufficient physical activity, and infections (3). Approximately 19% of incident cancers have been attributed to cigarette smoking, 7.8% to excess body weight, and 5.6% to alcohol consumption (3).

Given the rising cancer incidence rates among young women and the increasingly diverse and aging US population (1), we sought to determine trends in cancer incidence rates by race and ethnicity and age group during an 18-year period. We focused on cancers associated with 5 modifiable risk factors: tobacco use, excess body fat, alcohol consumption, insufficient physical activity, and human papillomavirus (HPV) infection (4).

## Methods

This cross-sectional study evaluated data from 2001 through 2018 from the US Cancer Statistics (USCS) database, which includes data from the Centers for Disease Control and Prevention’s National Program of Cancer Registries and the National Cancer Institute’s Surveillance, Epidemiology, and End Results (SEER) Program. Cancers associated with tobacco use, obesity (body mass index [BMI] ≥30.0, calculated as weight in kilograms divided by height in meters square), alcohol consumption, insufficient physical activity (<150 minutes of moderate-intensity or <75 minutes of vigorous-intensity aerobic activity weekly), and HPV infection were identified by predefined SEER*Stat variables (4). Our analysis of 28,175,859 cancers was limited to women aged 20 years or older diagnosed with cancers associated with the 5 aforementioned modifiable risk factors. We excluded cancers associated with other risk factors. Cancers were identified by International Classification of Disease for Oncology-3 site codes (5). We analyzed data by modifiable risk factor, age group (20–49 vs ≥50 y), and race and ethnicity: non-Hispanic White, non-Hispanic Black, Hispanic (all races), and non-Hispanic Asian or Pacific Islander. We used SEER*Stat version 8.3.8 (National Cancer Institute) to abstract data and Joinpoint regression program 4.8.0.1 (National Cancer Institute) to calculate trends. Age-specific incidence rates were crude rates by age group. We used annual percentage change (APC) with 95% CIs to describe trends in each segment and average annual percentage change (AAPC) with 95% CIs in the entire interval. Rates were considered to increase or decrease if *P* < .05. To characterize the incidence of cancer in each age group, we analyzed the total number of age-specific cancer cases.

## Results

During the study period, 11,198,521 cancers associated with the 5 modifiable risk factors were diagnosed in the US; 9,422,556 (84%) of these cancers were diagnosed among women aged 50 years or older, and 16% (n = 1,775,965) were diagnosed among women aged 20 to 49 years. The largest AAPC (1.58; 95% CI, 1.10–2.07; *P* < .001) was for obesity-associated cancers among women aged 20 to 49 years: incidence increased from 47.2 to 61.4 per 100,000 persons from 2001 to 2018, with the sharpest increase seen from 2001 to 2009. The only other significant increase was found for cancers associated with insufficient physical activity (AAPC, 1.05; 95% CI, 0.39–1.71; *P* = .002). HPV-associated cancers among women aged 20 to 49 years decreased from 13.8 to 11.6 per 100,000 persons from 2001 to 2018 (AAPC, −0.96; 95% CI, −1.74 to −0.17; *P* < .02). Trends in alcohol consumption– and tobacco use–associated cancers did not change (Table 1).

**Table 1:** Age-Specific Incidence and Trends in Modifiable Risk Factor–Associated Cancers Among US Women of All Races and Ethnicities Aged 20 Years or Older, Stratified by Age Group, United States Cancer Statistics, 2001–2018^a^

Risk factor, by age group, y	Age-specific incidence, 2001–2018	Trend 1		Trend 2		Trend 3		Trend 4		2001–2018
		Year	APC (95% CI)	Year	APC (95% CI)	Year	APC (95% CI)	Year	APC (95% CI)	AAPC (95% CI)
**Obesity-associated cancers**										
20–49	47.21 to 61.40	2001–2009	3.16^b^ (2.90 to 3.42)	2009–2012	0.50 (−1.64 to 2.68)	2012–2015	2.08 (−0.05 to 4.25)	2015–2018	−1.93^c^ (−2.95 to −0.89)	1.58^b^ (1.10 to 2.07)
≥50	755.46 to 688.87	2001–2004	−2.52^c^ (−3.81 to −1.20)	2004–2018	−0.08 (−0.20 to 0.04)	—	—	—	—	−0.52^b^ (−0.75 to −0.29)
**Alcohol consumption–associated cancers**										
20–49	84.27 to 84.85	2001–2008	0.58^b^ (0.35 to 0.80)	2008–2011	−1.33 (−2.96 to 0.32)	2011–2018	0.20 (−0.02 to 0.43)	—	—	0.09 (−0.20 to 0.37)
≥50	569.35 to 481.56	2001–2004	−3.48^b^ (−4.86 to −2.09)	2004–2011	−0.82^c^ (−1.29 to −0.34)	2011–2018	−0.01 (−0.37 to 0.34)	—	—	−0.96^b^ (−1.27 to −0.66)
**Tobacco use–associated cancers**										
20–49	42.36 to 41.61	2001–2007	1.06^c^ (0.61 to 1.52)	2007–2013	−1.54^b^ (−2.12 to −0.95)	2013–2016	1.42 (−1.27 to 4.17)	—	—	−0.14 (−0.65 to 0.39)
≥50	527.09 to 441.52	2001–2008	−1.12^b^ (−1.26 to −0.97)	2008–2011	−2.16^c^ (−3.22 to −1.10)	2011–2016	−0.13 (−0.46 to 0.21)	2016–2018	−1.26^c^ (−2.28 to −0.23)	−1.03^b^ (−1.24 to −0.82)
**Insufficient physical activity–associated cancers**										
20–49	14.98 to 18.06	2001–2008	1.16^c^ (0.69 to 1.64)	2008–2013	0.11 (−0.96 to 1.20)	2013–2016	4.59^c^ (1.23 to 8.06)	2016–2018	−2.17 (−5.28 to 1.04)	1.05^b^ (0.39 to 1.71)
≥50	567.99 to 502.58	2001–2004	−3.44^b^ (−4.81 to −2.05)	2004–2011	−0.42 (−0.89 to 0.05)	2011–2018	0.22 (−0.12 to 0.57)	—	—	−0.70^b^ (−1.00 to −0.39)
**Human papillomavirus–associated cancers**										
20–49	13.81 to 11.60	2001–2009	−0.73^c^ (−1.13 to −0.33)	2009–2013	−2.44^c^ (−4.31 to −0.54)	2013–2016	2.14 (−1.72 to 6.15)	2016–2018	−3.44 (−7.15 to 0.42)	−0.96^c^ (−1.74 to −0.17)
≥50	28.78 to 29.67	2001–2004	−1.33^c^ (−2.34 to −0.30)	2004–2016	0.92^b^ (0.79 to 1.05)	2016–2018	1.37 (−3.03 to 0.33)	—	—	0.25 (0 to 0.50)

Abbreviations: —, does not apply; AAPC, average annual percentage change; APC, annual percentage change.

a Analysis was limited to women aged ≥20 years diagnosed with cancers associated with the following 5 risk factors: tobacco use, excess body fat, alcohol consumption, insufficient physical activity, and human papillomavirus infection. Trends based on incidence were analyzed by using the Joinpoint Regression Program version 4.8.0.1. The AAPC is a summary measure of the trend over a prespecified fixed interval. AAPC was computed as a weighted average of the APC from the Joinpoint model, with the weights equal to the length of the APC interval.

b *P* < .01.

c *P* < .05.

Our data revealed racial and ethnic differences in incidence trends. Among women aged 20 to 49 years (Figure A), obesity-associated cancers rose most rapidly among Hispanic (AAPC, 2.86; 95% CI, 2.36–3.36; *P* < .001) and non-Hispanic Asian and Pacific Islander women (AAPC, 2.19; 95% CI, 1.73–3.65; *P* < .001), at nearly twice the rate of non-Hispanic White women (AAPC, 1.39, 95% CI, 1.11–1.67; *P* < .001). Non-Hispanic Black women had the smallest increase in obesity-associated cancers (AAPC, 0.96, 95% CI, 0.41–1.52; *P* = .001). Alcohol consumption–associated cancers rose among Hispanic (AAPC, 1.09, 95% CI, 0.79–1.38; *P* < .001) and non-Hispanic Asian and Pacific Islander (AAPC, 1.33, 95% CI, 1.15–1.51; *P* < .001) women but did not change among non-Hispanic Black or non-Hispanic White women. Tobacco use–associated cancers increased among Hispanic women (AAPC, 0.81, 95% CI, 0.41–1.21; *P* < .001), decreased among non-Hispanic Black women (AAPC −1.10, 95% CI, −1.62 to −0.57; *P* < .001), and did not change among non-Hispanic White or non-Hispanic Asian and Pacific Islander women.

**Figure:**
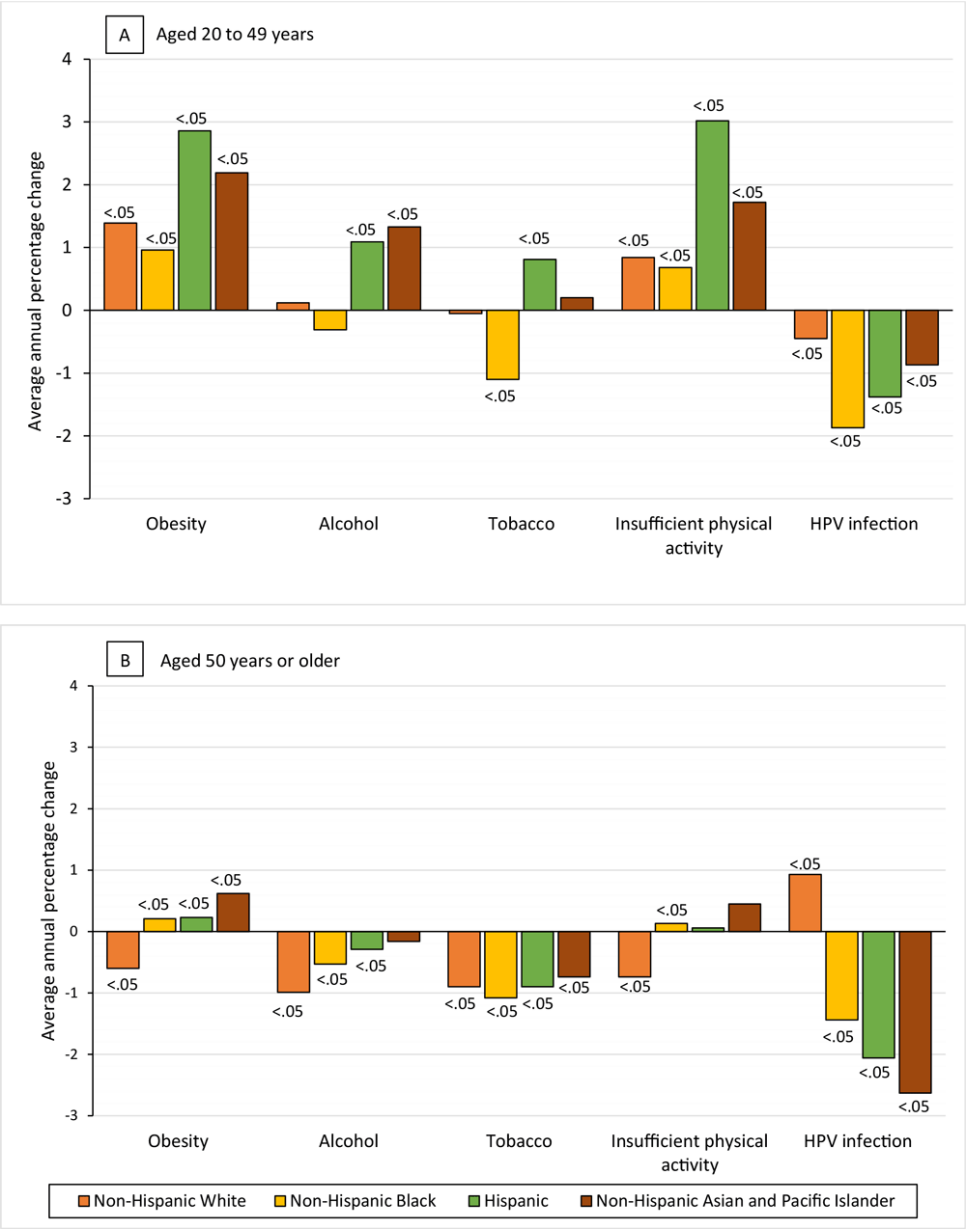
Average annual percentage change (AAPC) in modifiable risk factor–associated cancers among A) women aged 20 to 49 years and B) women aged 50 years or older, US Cancer Statistics database, 2001–2018. Values on bars indicate *P* values. Abbreviation: HPV, human papillomavirus.

**Table:** 

Race and ethnicity	Obesity	Alcohol	Tobacco	Insufficient physical activity	HPV infection
**A. Women aged 20 to 49 years, AAPC (95% CI) [*P* value]**					
Non-Hispanic White	1.39 (1.11 to 1.67) [<.05]	0.12 (–0.27 to 0.51)	−0.05 (–0.77 to 0.67)	0.84 (0.06 to 1.62) [<.05]	−0.45 (–0.69 to –0.21) [<.05]
Non-Hispanic Black	0.96 (0.41 to 1.52) [<.05]	−0.31 (–0.63 to 0)	−1.10 (−1.62 to −0.57) [<.05]	0.68 (0.37 to 0.99) [<.05]	−1.87 (–2.14 to –1.59) [<.05]
Hispanic	2.86 (2.36 to 3.36) [<.05]	1.09 (0.79 to 1.38) [<.05]	0.81 (0.41 to 1.21) [<.05]	3.02 (2.44 to 3.61) [<.05]	−1.38 (–2.18 to –0.57) [<.05]
Non-Hispanic Asian and Pacific Islander	2.19 (1.73 to 3.65) [<.05]	1.33 (1.15 to 1.51) [<.05]	0.20 (–0.03 to 0.43)	1.72 (1.32 to 2.13) [<.05]	−0.87 (–1.51 to –0.23) [<.05]
**B. Women 50 years or older, AAPC (95% CI) [*P* value]**					
Non-Hispanic White	−0.60 (−0.83 to −0.36) [<.05]	−0.99 (−1.30 to −0.67) [<.05]	−0.90 (–1.11 to –0.67) [<.05]	−0.74 (−1.04 to −0.44) [<.05]	0.93 (0.60 to 1.26) [<.05]
Non-Hispanic Black	0.21 (0.03 to 0.40) [<.05]	−0.53 (−0.93 to −0.12) [<.05]	−1.08 (–1.17 to –1.00) [<.05]	0.13 (0.03–0.24) [<.05]	−1.44 (−1.73 to −1.15) [<.05]
Hispanic	0.23 (0.09 to 0.38) [<.05]	−0.29 (−0.53 to −0.06) [<.05]	−0.90 (–1.24 to –0.55) [<.05]	0.06 (–0.20 to 0.33)	−2.06 (−2.51 to −1.60) [<.05]
Non-Hispanic Asian and Pacific Islander	0.62 (0.46 to0.78) [<.05]	−0.16 (–0.64 to 0.32)	−0.74 (–0.89 to –0.60) [<.05]	0.45 (–0.05 to 0.95)	−2.63 (−4.25 to −0.98) [<.05]

Among women aged 50 years or older (Figure B), tobacco use–associated cancers decreased among all ethnic and racial groups, and alcohol consumption–associated cancers decreased among non-Hispanic White (AAPC, −0.99; 95% CI, −1.30 to −0.68; *P* < .001), non-Hispanic Black (AAPC, −0.53; 95% CI, −0.93 to −0.12; *P* = .01), and Hispanic women (AAPC, −0.29; 95% CI, −0.53 to −0.06; *P* = .02) but did not change among non-Hispanic Asian and Pacific Islander women. Obesity-associated cancers decreased only among non-Hispanic White women (AAPC, −0.60; 95% CI, −0.83 to −0.36; *P* < .001); these cancers increased among non-Hispanic Black, Hispanic, and non-Hispanic Asian and Pacific Islander women, with the greatest increase among non-Hispanic Asian and Pacific Islander women (AAPC, 0.62; 95% CI, 0.46–0.78; *P* < .001). Similarly, cancers associated with insufficient physical activity decreased among non-Hispanic White women (APC, −0.74; 95% CI, −1.04 to −0.44; *P* < .001), increased among non-Hispanic Black women (AAPC, 0.13; 95% CI, 0.03 to 0.24; *P* = .02), and did not change among Hispanic and non-Hispanic Asian and Pacific Islander women. HPV infection–associated cancers decreased among non-Hispanic Black (AAPC, −1.44, 95% CI, −1.73 to −1.15; *P* < .001), Hispanic (AAPC, −2.06; 95% CI, −2.51 to −1.60; *P* < .001), and non-Hispanic Asian and Pacific Islander women (AAPC, −2.63; 95% CI, −4.25 to −0.98; *P* = .002).

We found similarities in cancer incidence trends for the most common cancer types in both age groups. However, breast cancer accounted for a greater proportion of cancers among younger women than older women (45.0% vs 34.0%) as did thyroid (14.8% vs 2.8%) and cervical cancers (6.6% vs 1.2%) (Table 2).

**Table 2:** Cases of Modifiable Risk Factor–Associated Cancers Among US Women of All Races and Ethnicities Aged 20 Years or Older, Stratified by Age Group, United States Cancer Statistics, 2001–2018

Cancer type	No. (%)	
	Women aged 20–49 years (n = 1,775,965)	Women aged ≥50 years (n = 9,422,556)
Acute myeloid leukemia	18,397 (1.0)	82,486 (0.9)
Anal and rectal squamous cell carcinoma	10,253 (0.6)	57,772 (0.6)
Cervix uteri	117,023 (6.6)	112,751 (1.2)
Colon and rectum	124,086 (7.0)	1,142,756 (12.1)
Corpus and uterus not otherwise specified	107,759 (6.1)	735,174 (7.8)
Esophagus	3,305 (0.2)	59,835 (0.6)
Female breast	798,605 (45.0)^a^	3,206,778 (34.0)^b^
Gallbladder	2,697 (0.2)	43,689 (0.5)
Gastric cardia	2,372 (0.1)	24,742 (0.3)
Kidney	50,317 (2.8)	306,478 (3.2)
Larynx	4,899 (0.3)	41,132 (0.4)
Lip, oral cavity, and pharynx	30,551 (1.7)	175,233 (1.9)
Liver	6,803 (0.4)	95,093 (1.0)
Meningioma	730 (0.04)	3,246 (0.03)
Multiple myeloma	11,051 (0.6)	156,691 (1.7)
Oropharyngeal squamous cell carcinoma	6,907 (0.4)	47,708 (0.5)
Ovary	69,151 (3.9)	314,297 (3.3)
Pancreas	18,837 (1.1)	349,564 (3.7)
Stomach	18,269 (1.0)	137,278 (1.5)
Thyroid	263,292 (14.8)	259,595 (2.8)
Trachea, lung, and bronchus	83,421 (4.7)	1,712,034 (18.2)
Urinary bladder	14,641 (0.8)	292,400 (3.1)
Vaginal squamous cell carcinoma	1,784 (0.1)	12,621 (0.1)
Vulvar squamous cell carcinoma	10,815 (0.6)	53,203 (0.6)

a Premenopausal breast cancer.

b Postmenopausal breast cancer.

## Discussion

We provided data on cancers associated with 5 modifiable risk factors for nearly the entire US female population aged 20 years or older from 2001 through 2018. Risk factor–associated cancers increased among women aged 20 to 49 years, with obesity-associated cancers rising most rapidly. Conversely, we found decreasing trends in cancer incidence among women aged 50 or older, with the largest decreases in tobacco use–associated cancers.

Several studies reported similar findings for increasing trends in obesity-associated cancers. Almost one-tenth (9.5%) of cancers in the US can be attributed to obesity (6). Sung et al observed that obesity-associated cancers rose more rapidly among adults aged 25 to 49 years than among adults aged 50 years or older (7). Our study supports that obesity-associated cancers are increasing more rapidly among younger women than older women. By stratifying our analysis by race and ethnicity, we discovered that obesity-associated cancers are increasing among younger women across all races and ethnicities. We found the greatest increase among Hispanic women, nearly 3 times the rate of non-Hispanic Black women and twice the rate of non-Hispanic White women. Obesity-associated cancers did not increase among non-Hispanic Black women, as reflected elsewhere (8).

Tobacco use accounts for 35% of preventable cancers among women (3). Previous studies reported decreasing incidence rates for tobacco use–associated cancers (9,10). However, our study found that among younger women, tobacco use–associated cancers increased among Hispanic women but did not change among non-Hispanic White or non-Hispanic Asian and Pacific Islander women, and they decreased among older women. Although tobacco use in the US has decreased, a disparity persists between tobacco use–associated cancers diagnosed among younger and older women, and the impact of smokeless tobacco use should be further explored (11,12).

Our study lacks data on individual exposures, and we did not account for cancers associated with more than 1 modifiable risk factor. Our data may underrepresent the proportions of cancers associated with the 5 risk factors examined. Accounting for the cumulative effects of these risk factors over time as they pertain to cancer risk is difficult. Nevertheless, we determined nationally representative trends and incidence estimates.

Our study showed that the incidence of modifiable risk factor–associated cancers is trending downward among older women but upward among younger women. The incidence of obesity–associated cancers is rising rapidly, with larger increases among younger women and Hispanic women. Studies that examine the effect of obesity on cancers diagnosed in these populations are warranted.
